# RNA/DNA ratios in American glass eels (*Anguilla rostrata*): evidence for latitudinal variation in physiological status and constraints to oceanic migration?

**DOI:** 10.1002/ece3.212

**Published:** 2012-05

**Authors:** Simon Laflamme, Caroline Côté, Pierre-Alexandre Gagnaire, Martin Castonguay, Louis Bernatchez

**Affiliations:** 1 IBIS (Institut de Biologie Intégrative et des Systèmes), Université LavalQuébec, QC, G1V 0A6, Canada; 2 Institut Maurice-Lamontagne, Ministère des Pêches et des Océans850 Route de la Mer, Mont-Joli, QC, G5H 3ZH, Canada

**Keywords:** Eel, fish, migration, phenotypic plasticity, RNA/DNA ratio

## Abstract

During their larval leptocephalus phase, newly hatched American eels undergo an extensive oceanic migration from the Sargasso Sea toward coastal and freshwater habitats. Their subsequent metamorphosis into glass eel is accompanied by drastic morphological and physiological changes preceding settlement over a wide geographic range. The main objective of this study was to compare RNA/DNA ratios and condition factor among glass eels in order to test the null hypothesis of no difference in physiological status and metabolic activity of glass eels at the outcome of their oceanic migration. This was achieved by analyzing glass eel samples collected at the mouth of 17 tributaries covering a latitudinal gradient across the species distribution range from Florida to Gaspésie (Québec). Our main observations were (i) a latitudinal increase in mean total length; (ii) a latitudinal variation in mean RNA/DNA ratios, which was best explained by a quadratic model reaching its minimum in the central range of sampling locations; and (iii) a latitudinal variation in Fulton's condition factor, which was best explained by a quadratic model reaching its maximum in the central range of sampling locations. Below we discuss the possible links between latitudinal variation in glass eel physiological status and variable energetic and environmental constraints to oceanic migration as a function of latitudinal distribution.

## Introduction

To complete its life cycle, the American eel (*Anguilla rostrata*) must migrate 1.5–5 thousands km from the American East coast to the Sargasso Sea in the Atlantic Ocean (Schmidt 1923). Reproduction is semelparous and newly hatched leptocephali larvae undertake the reverse itinerary to reach, approximately six months to one year later, the continental waters for the growing phase of their life cycle (Wang and Tzeng 1998; Arai et al. 2000; Bonhommeau et al. 2010). Fundamental physiological changes mark the metamorphosis from leptocephalus to the glass eel phase (Powles et al. 2006), which precedes the colonization of coastal, estuarine, and freshwater habitats (Lecomte-Finiger 1994; Jessop et al. 2002). The life cycle is completed about 3–20 years later when full-grown mature silver eels migrate back to the Sargasso Sea to breed and die.

The American eel also exhibits different generalist strategies: the species is found in both brackish and freshwater habitats over a wide geographical range that spreads from northern South America to southern Greenland (Tesch 1977). Although the null hypothesis of panmixia has not been rejected based on previous genetic studies using neutral markers (Avise et al. 1986; Wirth and Bernatchez 2003), locally adaptive alleles segregating within the species’ gene pool might partly account for its ability to colonize such an heterogeneous habitat (Williams et al. 1973; Koehn and Williams 1978; Gagnaire et al. 2012). Moreover, quantitative genetic differences for growth between eels from different localities have also been revealed by controlled experiments (Côté et al. 2009). Nevertheless, the potential of eels to occupy a wide range of habitats has generally been attributed to a strong phenotypic plasticity (Edeline 2007). Several factors such as population density, proximity to the Sargasso Sea, and prey abundance have variously been singled out as key factors potentially influencing eel phenotypic variability among locations (Holmgren et al. 1997; Davey and Jellyman 2005; Vélez-Espino and Koops 2009).

One phenotype in which eels from different localities differ very early on at the larval phase is growth rate. Based on otolith aging, Wang and Tzeng (1998) revealed that the arrival site of glass eels impacts upon their postrecruitment growth rate, with southern glass eels growing more rapidly than northern ones. Wang and Tzeng (1998) also showed that glass eels captured from the middle of the species’ range (approximately the Cheasapeake Bay area) had a shorter leptocephalus phase associated with a faster growth rate during that stage relative to glass eels captured from either geographical extremity along the Atlantic coast. However, these results must be interpreted cautiously since analyses based on otolith aging to infer growth have been criticized due to recurring discrepancies in the count of daily growth increments (McCleave 2008; Fukuda et al. 2009; Bonhommeau et al. 2010).

The main objective of this study was to compare RNA/DNA ratio and condition factor among glass eels from different locations in order to test the null hypothesis of no difference in physiological status, and metabolic activity at the outcome of their oceanic migration. This was achieved by analyzing glass eel samples collected at the mouth of 17 tributaries covering a wide latitudinal gradient across the species distribution range extending from Florida (USA) to Gaspésie (Québec, Canada). Condition factor has been widely used as a standard measure of general physiological condition in fishes, including in eels (Simon 2007). For the last 30 years, RNA/DNA ratio has been correlated with nutritional condition in larval and juvenile fishes and larval fish growth rate in the sea (Buckley 1984). By tuning in the pace of protein synthesis in the cell, the amount of ribosomic, messenger, and transfer RNAs provides information on the metabolic status of the whole organism. RNA quantity is standardized by the DNA content, which is essentially proportional to the number of cells the organism contains, to provide an estimation of the organism's metabolic rate, which can be used to assess its energetic status (Chícharo and Chícharo 2008). Thus, under controlled experimental settings, fish that were regularly fed displayed an increased RNA/DNA ratio compared with minimally fed fish (Buckley 1979, 1984; Clemmesen 1994). This correlation may also hold in the wild, although additional variables must be accounted for in results interpretation (Chícharo et al. 1998; Fonseca et al. 2006). This study represents the first attempt to interpret regional variation in the physiological status of early life stages in Atlantic eels by means of RNA/DNA ratio.

## Materials and Methods

### Glass eels collection

**Figure 1:**
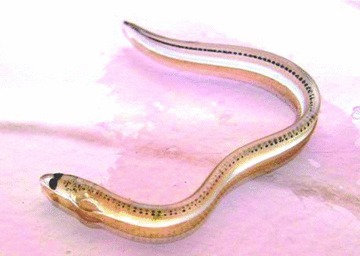
Young glass eel collected in June 2008 at the Grande-Rivière-Blanche sampling site. (Photo credit: Guy Verreault).

**Figure 2:**
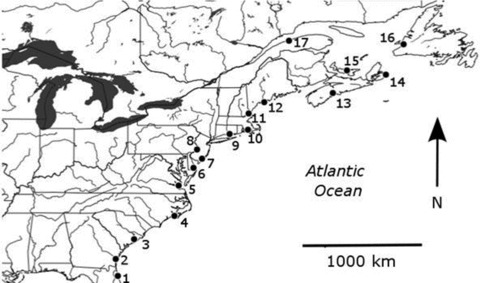
Map of eastern North America showing positions of sample locations (see Table 1 for sampling details).

Between January and July 2008, glass eels at pigmentation stage V–VI A2 (Élie et al. 1982; Fig. 1) were collected by local scientific crews with dip nets at the mouth of 17 rivers distributed along the North American east coast (Table 1; Fig. 2). In all instances, glass eels belonged to the early recruitment wave of the season. In order to minimize the influence of circadian variations on the RNA/DNA ratio (Chícharo et al. 2001), all individuals were captured at night (same period of the day). Total body length (measured from head to tail) was measured to the nearest millimeter (mm) and total weight measured to the nearest milligram (mg) before being placed immediately in a nontoxic salt-saturated storage solution (RNAlater® Ambion, Austin, TX) and stored for a minimum of 48 h at 4°C to ensure the complete desiccation of the samples, which enables to preserve RNA and DNA integrity at room temperature for safe shipping.

**Table 1:** List of localities sampled (location number on Fig. 2), date of capture, sample size, mean total length (±standard deviation), and Fulton's condition factor (*K*) (±standard deviation) of glass eels collected from 17 locations. NA, data not available.

Sample location	Latitude Longitude	Date of capture (mm–yy)	*n*	Length (mm)	*K*
Guana River Dam, Florida (1)	30°02′N–81°33′W	01–08	10	51 ± 3	0.704 ± 0.229
Mornings-AR & Gabes, Georgia (2)	31°31′N–81°47′W	02–08	10	50 ± 3	0.710 ± 0.208
Cooper River, South Carolina (3)	32°93′N–80°01′W	02–08	10	52 ± 3	0.743 ± 0.227
Black Creek, North Carolina (4)	34°77′N–76°81′W	02–08	10	54 ± 2	NA
Wormley Creek, Virginia (5)	37°22′N–76°49′W	03–08	10	57 ± 4	0.677 ± 0.188
Millsboro Pond Spillway, Delaware (6)	38°59′N–75°29′W	02–08	10	58 ± 4	0.908 ± 0.188
Patcong Creek Linwood, New Jersey (7)	39°36′N–74°58′W	04–08	10	54 ± 3	0.808 ± 0.215
Poquessing Creek, Pennsylvania (8)	40°05′N–74°98′W	04–08	10	56 ± 2	0.853 ± 0.114
Taylor River & Old Saybrook, Connecticut (9)	41°30′N–72°40′W	05–08	10	56 ± 1	0.753 ± 0.140
Parker River, Massachussetts (10)	41°68′N–70°92′W	04–08	10	67 ± 4	0.772 ± 0.252
Taylor River, New Hampshire (11)	42°93′N–70°86′W	04–08	10	57 ± 3	0.696 ± 0.214
Boothbay Harbor, Maine (12)	43°84′N–69°65′W	04–08	10	57 ± 4	0.612 ± 0.237
East River, Nova Scotia (13)	44°59′N–64°17′W	04–08	5	60 ± 3	NA
Mira River, Nova Scotia (14)	46°04′N–59°97′W	07–08	5	61 ± 3	0.578 ± 0.250
Rustico Bay, Prince-Edward-Island (15)	46°41′N–63°04′W	07–08	10	61 ± 3	NA
Codroy Bay, Newfoundland (16)	48°47′N–58°52′W	07–08	10	60 ± 2	NA
Grande-Rivière-Blanche, Québec (17)	48°78′N–67°69′W	06–08	10	63 ± 4	0.676 ± 0.344

### RNA/DNA ratio measures

For each sampled locality, except two (Table 1), RNA/DNA ratio was quantified for 10 randomly selected glass eels. Glass eels were sliced in four sections and rapidly immerged in vials containing a solution of 990 μL of lysis buffer (RNA Mini Kit, Invitrogen, Carlsbad, CA) and 10 μL 2-mercaptoethanol (Sigma-Aldrich, St.Louis, MO) that allowed preserving both RNA and DNA integrity for simultaneous extraction. Samples were individually grounded by inserting a metal bead in each sample vial, which were then placed in TissueLyser II (QIAGEN, Dusseldorf, Germany) for 10 cycles of 1 min at 25 Hz. Eel homogenates were stored at –80°C until nucleic acid extraction.

### Nucleic acids extraction and quantification

DNA and RNA extraction was carried out simultaneously with an N-lauroylsarcosine protocol adapted from Caldarone and Buckley (1991). After thawing, 50 μL of the eel homogenate solution was transferred in a second vial to which 50 μL of a 2%*N*-lauroylsarcosine (Sigma-Aldrich, Steinheim, Germany) solution (2 g dissolved in 100 mL TE buffer) was added. The vial was vortexed for 30 sec, and 400 μL of TE buffer was subsequently added to reach a 0.2%*N*-lauroylsarcosine final concentration. To precipitate proteins, the vial was then gently shaken and centrifuged for 15 min at 14,000 *g*. A total of 100 μL of the supernatant was then transferred in a third vial already containing 900 μL of TE buffer. Finally, 25 μL of the well-mixed 1:10 dilution was used for spectrofluorometrical RNA and DNA measurements.

Nucleic acids quantification protocol was adapted from Caldarone et al. (2001). Quantification was performed using the SYBR® Gold Nucleic Acid Gel Stain dye (Invitrogen). Spectrofluorometry readings were carried out in 96-well microplates at 25°C with a Fluoroskan Ascent FL (Thermolabsystems, Helsinki, Finland) set for 485 nm excitation wavelength and 527 nm emission wavelength. Microplate wells were filled with 75 μL of 1× SYBR Gold solution before 25 μL of the 1:10 nucleic acid dilution (or TE buffer blank, or DNA standard, or RNA standard) was added in each. The microplate was agitated for 5 min and put at rest for another 5 min for optimal dye–nucleic acid binding. A first series of readings was carried out in order to measure the combined RNA + DNA signal. Following this step, 5 μL of 33 µg/mL RNase A (Roche Diagnotics, Bale, Swiss) was added to each well to eliminate the signal due to RNA alone. The microplate was agitated for 5 min and put at rest for 15 min for complete RNase digestion. A second series of readings was then carried out to measure the signal due to DNA alone. Signal due to RNA alone was calculated as the difference between first and second readings (Caldarone et al. 2001). We empirically determined that an additional DNA digestion step was not necessary because the background signal due to proteins was nonsignificant (<10%) (data not shown). Hence, signal due to DNA alone was directly inferred from the second reading. In order to convert spectrofluorometer fluorescent readings to DNA and RNA concentrations, DNA and RNA standards (veal thymus DNA and 18S and 28S veal liver RNA, Sigma-Aldrich) were used in parallel with each experiment and standards data were pooled to construct calibration curves.

### Sensitivity of the quantification method

Nucleic acid extraction and quantification was repeated 12 times on homogenates coming from a single glass eel (systematic use of replicate controls in parallel with real samples). This allowed estimating the sensitivity of the spectrofluorometry quantification method. DNA measurements carried out on controls showed a coefficient of variation (CV) of 3.4%. However, RNA measurements carried out on those same replicates showed a higher CV of 19.2%, which contributed to increase variance on RNA/DNA ratio estimates and therefore reduced the strength of the observed correlation between RNA/DNA ratios with latitude (see results).

### Data analysis

Fulton's condition factor (*K*) was calculated from the total weight (g) and length (cm) of the eels using the formula of Fulton (1904). This index could not be estimated for four of the 17 sampling sites because fresh weight was not measured (Table 1). To test the null hypothesis of geographical uniformity of RNA/DNA ratios and condition factor, linear and quadratic regressions between these variables and latitude data were performed. Here, latitude is considered as a proxy for other variables that may have an effect on RNA/DNA ratio and condition factor (e.g., temperature, food abundance, migrational distance, age and size of larvae at metamorphosis, etc.) but for which reliable data are not available. In both cases, quadratic regression model was retained as it better fits the data than linear regression, based on both the level of correlation and dispersion of residues. Linear and quadratic regression models were also applied to relate RNA- and DNA-alone concentrations with latitude. Due to multicolinearity among latitude, date of capture, and body length, a multiple regression model could not be applied. A Tukey test was applied to all localities except two with small sample sizes (Table 1) in order to identify homogenous groupings of localities characterized by RNA/DNA ratios that significantly differed from other such groupings. All statistical tests were carried out with SAS software (version 9.01, SAS institute Inc., Cary, NC) in compliance with normality and homogeneity of variances precept.

## Results

### Glass eel body length

**Figure 3:**
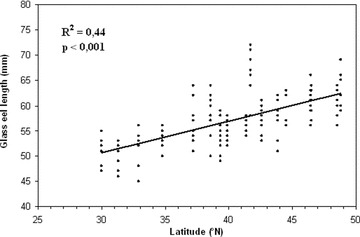
Distribution of glass eel total length (in mm) as a function of latitudinal distribution.

Mean glass eel length varied from 50 ± 3 mm for the Georgia sample (31.3°N) up to 67 ± 4 mm for the sample from Massachusetts (41.7°N) (Table 1). Linear regression revealed a highly significant positive correlation between latitude and body length (Length [mm] = 0.063 × lat + 3.18; *r*^2^= 0.44, *P*-value < 0.0001) (Fig. 3). The correlation coefficient increased to *r*^2^= 0.54 when the Massachusetts sample, with a particularly large value of body length given its latitude, was excluded from the regression.

### RNA/DNA ratios and condition factor

RNA concentration was positively correlated with latitude, which was best predicted by a linear regression model (RNA [mg/individual] = 0.00096 × lat + 0.0155; *r*^2^= 0.15; *P*-value < 0.0001). The mean overall RNA concentration was 0.22 ± 0.04 mg/individual, varying from a minimum of 0.16 ± 0.02 mg in the Florida sample to a maximum of 0.31 ± 0.05 mg in the Gaspésie sample (Table 2). Conversely, the model that best described observed DNA concentration as a function of latitude was an inverse quadratic regression (*r*^2^= 0.18, *P*-value < 0.0001), with a maximal DNA concentration predicted at 40.3°N. The mean overall DNA concentration was 1.09 ± 0.32 mg/individual. The Pennsylvania sample, which lies approximately in the center of the sampled geographical range, displayed an average DNA concentration (2.10 ± 0.17 mg/individual) that was much higher than any other sampled locality (Table 2). Even when this sample was excluded, the inverse quadratic model still significantly and best explained the latitudinal pattern of variation in DNA concentration (*r*^2^= 0.14; *P*-value < 0.0001).

**Table 2:** Mean RNA concentration, DNA concentration, and RNA/DNA ratio ± standard deviation for each sampled location. Localities sharing the same letter code did not differ significantly in their mean RNA/DNA ratios based on a Tukey test. Both samples from Nova Scotia (12, 13) were not included in the Tukey test because of too small sample sizes (see Table 1). Sample locations sharing the same capital letter are not significantly different one from the other.

Sample location	RNA (mg/individual)	DNA (mg/individual)	RNA/DNA ratio	
Grande Rivière Blanche, Québec (17)	0.31 ± 0.05	0.95 ± 0.32	0.344 ± 0.07	A
Parker River, Massachussetts (10)	0.20 ± 0.03	0.85 ± 0.19	0.252 ± 0.08	B
Prince Edward Island (15)	0.25 ± 0.06	1.05 ± 0.26	0.250 ± 0.05	B
Cape Breton, Nova Scotia (14)	0.21 ± 0.02	0.85 ± 0.08	0.250 ± 0.04	-
Boothbay Harbor, Maine (12)	0.24 ± 0.03	1.01 ± 0.11	0.237 ± 0.03	BC
Guana River Dam, Florida (1)	0.16 ± 0.02	0.72 ± 0.09	0.229 ± 0.03	BC
Cooper River, South Carolina (3)	0.22 ± 0.03	1.00 ± 0.10	0.227 ± 0.04	BC
Patcong Creek Linwood, New Jersey (7)	0.20 ± 0.05	0.93 ± 0.05	0.215 ± 0.05	BC
Taylor River, New Hampshire (11)	0.25 ± 0.05	1.15 ± 0.12	0.214 ± 0.04	BC
Georgia (2)	0.17 ± 0.03	0.84 ± 0.07	0.208 ± 0.04	BCD
Newfoundland (16)	0.21 ± 0.04	1.11 ± 0.09	0.189 ± 0.04	BCD
Wormley Creek, Virginia (5)	0.26 ± 0.05	1.40 ± 0.13	0.188 ± 0.04	BCD
Millsboro Pond Spillway, Delaware (6)	0.18 ± 0.02	0.95 ± 0.13	0.188 ± 0.02	BCD
East River, Nova Scotia (13)	0.17 ± 0.03	0.93 ± 0.10	0.183 ± 0.03	-
Black Creek, North Carolina (4)	0.18 ± 0.05	1.09 ± 0.14	0.170 ± 0.07	CDE
Taylor River—Old Saybrook, Connecticut (9)	0.19 ± 0.04	1.38 ± 0.22	0.140 ± 0.02	DE
Poquessing Creek, Pennsylvania (8)	0.24 ± 0.04	2.10 ± 0.17	0.114 ± 0.02	E

**Figure 4:**
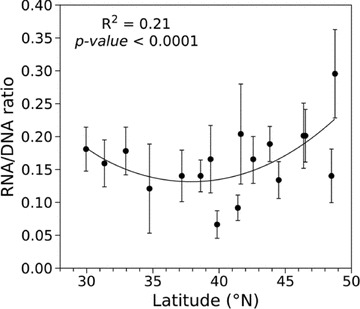
Mean RNA/DNA ratios (with standard deviation) as a function of latitudinal distribution.

Given the above RNA and DNA concentrations, the mean RNA/DNA ratios ranged from 0.114 in Pennsylvania up to 0.344 in Gaspésie, Québec (Table 2). A quadratic model best explained the variance in RNA/DNA ratio as a function of latitude (ratio = 0.00080 × lat^2^– 0.0608 × lat + 1.331, *r*^2^= 0.21, *P*-value < 0.0001). The negative parabolic regression curve reached its minimum at about 38.0°N corresponding to a predicted RNA/DNA ratio of 0.18 (Fig. 4). The highest RNA/DNA ratio was observed for the northernmost sample (Gaspésie, Québec), which was also the only location significantly higher than any other single locality (RNA/DNA ratio = 0.344 ± 0.07, Tukey test; *P*-value = 0.001) (Table 2). Mean ratios for all other localities varied between 0.114 ± 0.02 in Pennsylvania and 0.252 ± 0.08 in Massachusetts.

**Figure 5:**
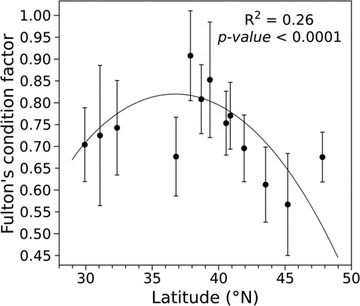
Mean condition factor (with standard deviation) as a function of latitudinal distribution.

Mean Fulton's condition factor ranged from 0.578 in Maine up to 0.908 in Delaware (Table 1). A positive quadratic model best explained the variance in condition factor (*K*) as a function of latitude (*K*= 0.760563 – 1.149280 × lat – 2.267006 × lat^2^, *r*^2^= 0.26, *P*-value < 0.0001). The positive parabolic regression curve reached its maximum at about 37.0°N corresponding to a predicted condition factor of about 0.82 (Fig. 5). Thus, the relationship between Fulton's condition factor versus latitude was essentially the inverse of that observed for RNA/DNA ratio.

## Discussion

The main objective of this study was to compare RNA/DNA ratio and condition factor among glass eels in order to test the null hypothesis of no latitudinal difference in physiological status and metabolic activity at the end of their oceanic migration and prior the freshwater phase of their life cycle. This was achieved by analyzing glass eel samples collected at the mouth of 17 tributaries covering a wide latitudinal gradient across the species distribution range extending from Florida to Québec. Our main observations were (i) a latitudinal increase in mean total length as previously reported by Wang and Tzeng (1998, 2000) on glass eels sampled 15 years ago; (ii) a significant latitudinal variation in mean RNA/DNA ratios, which was best explained by a quadratic model reaching its minimum in the central range of sampling locations; (iii) a significant latitudinal variation in mean Fulton's condition factor, which was best explained by a quadratic model reaching its maximum in the central range of sampling locations; and (iv) the highest RNA/DNA ratio observed for the Grand-Rivière-Blanche sample (Québec), which was also significantly higher than any other locality. Below we discuss the relevance of these observations regarding latitudinal variation in glass eel physiological status and its possible link with the variable environmental and energetic constraints to oceanic migration from the Sargasso Sea as a function of latitudinal distribution.

### RNA/DNA ratio as a proxy for growth rate

RNA/DNA ratio measured on fish larvae has routinely been linked to growth rate (i.e., Buckley 1979, 1984; Robinson and Ware 1988; Caldarone et al. 2003; Vinagre et al. 2008). Here, the observed ratio was the highest in Québec, where growth rate should paradoxically be expected to be the lowest due to latitude, and as suggested by the results of Wang and Tzeng (1998, 2000) and Côté et al. (2009) experimentally. This apparent contradiction might ensue from a complex multiparametric relationship linking growth rate to RNA/DNA ratio. Namely, a meta-analysis by Buckley et al. (2008) has demonstrated that the growth rate inferred from RNA/DNA ratio is strongly correlated to temperature. Therefore, and since the kinetics of protein synthesis both depends on the temperature and the amount of available RNA, interpreting variations in RNA/DNA ratio as indications of growth rate can be confounded by temperature variation across the sampling range. To get a quantitative sense of the differences involved, standard curves must be set up for the species under focus. In the current experiment, water temperature at the moment of capture was not recorded. However, from the Florida sampling site in January to Québec in July, larvae have most certainly been exposed very distinct thermal regime depending on location. For instance, NASA oceanic surveys from 2009 (NASA 2009) provide surface temperatures that remained relatively warm (over 18°C) in the midst of winter off the coast of Florida, while glass eels penetrating into the St. Lawrence Gulf between May and July would have been exposed to much colder temperatures (below 10°C). Even if precise temperature data were available, the lack of standard curves linking the RNA/DNA ratio to the growth rate for different temperatures precludes any robust inference of growth rate from RNA/DNA ratio measurements alone. Also, the RNA/DNA ratio reflects a global level of metabolic activity and growth may not be a major metabolic component during the glass eel phase (Lecomte-Finiger 1994). Below, we explore alternative interpretations for the observed patterns of RNA/DNA ratios.

### RNA/DNA ratio and condition factor as proxies for physiological stress associated with larval dispersal

In fish, RNA/DNA ratio values lower than 2 have usually been associated to prolonged fasting and an enhanced risk of mortality (Chícharo and Chícharo 2008). Here, none of the ratios measured exceeded 0.5. However, very low RNA/DNA ratios (between 0.6 and 1.1) have also been previously reported by Kawakami et al. (1999) for glass eels of the closely related Japanese eel (*A. japonica*). The apparently weak metabolic activity found in glass eel could be a characteristic of the growth strategy employed in fishes with a leptocephalus larval phase. For almost one year, the leptocephalus larva of American eel must ally survival and locomotion faculties while limiting its metabolic expenditures in order to successfully complete its oceanic journey (Schmidt 1923; Kettle and Haines 2006). Namely, it is believed that locomotion faculties are increased by the laterally compressed shape of leptocephali, which allows it to drift at minimal costs through oceanic currents (Castonguay and McCleave 1987; McCleave et al. 1998). As for growth, which is achieved during early development by stocking of acellular mass (mostly water), it represents a relatively small proportion of total metabolism, which varies between 4% and 39% of total metabolism (Bishop and Torres 2001). Lipids amount to 80% of the energy content and are thought to be synthesized via absorption of suspended particular matter in the water (Donnelly et al. 1995; Pfeiler 1996). At the time of their entry in estuaries, newly metamorphosed glass eels feed minimally and resort to their lipid reserves that they gradually break down (Lecomte-Finiger 1994; Sullivan et al. 2009). Similarly, Kawakami et al. (1999) observed in Japanese eel that endogenous lipid contents gradually waned during the period of estuarine entry, with glass eels arriving late exhibiting lower stocking levels. More recently, Gagnaire et al. (2012) provided evidence for spatially varying selection acting on allelic variation at the Acyl-carrier protein gene among the same glass eels that were analyzed in this study. Interestingly, this gene is directly involved in the fatty acid synthesis pathway, and thus, suggesting that the selective agent acting its allelic variation could relate to differential energetic constraints encountered by glass colonizing tributaries along the Atlantic coast.

Differential physiological stress associated with variable energetic constraints faced by *A. rostrata* larvae during their dispersal could in turn influence pattern of survival and recruitment in the species’ distribution range. Although catch per unit effort (CPUE) data are not available throughout their whole geographic range, the peak of recruitment apparently lies somewhere between Virginia to the South (Vélez-Espino and Koops 2009) and southern of Nova-Scotia to the North (Jessop 1998). Offshore, the most favorable oceanic conditions for glass eels (mild temperatures, proximity of Gulf Stream to the continent) possibly lies between Virginia and Massachusetts, also corresponding with the area of most abundant catches at sea (Kleckner and McCleave 1985). Indeed, more southeastern locations, although being also relatively close to the Sargasso Sea, exhibit lower levels of recruitment (Vélez-Espino and Koops 2009). The northward flowing Gulf Stream may likely keep glass eels from massively recruiting to the southern reaches of the North American coast (Kettle and Haines 2006). As for more northern locations, and in particular in the Gulf of St. Lawrence, which encompasses the three northernmost sampled localities, the southward flowing Labrador stream keeps waters cold all year around (Dutil et al. 2009). Dutil et al. (2009) also described constraints potentially encountered by glass eels in the Laurentian Channel (depth > 400 m), through which they must pass to enter the Gulf. Between 100 and 200 m depth, water temperatures constantly hover around the freezing point and beneath that depth, waters are severely oxygen-depleted. Among other things, such barriers hamper the normal use of selective tidal stream transport by eels in the Gulf of St. Lawrence (Edeline et al. 2004; Bureau Du Colombier et al. 2009). Moreover, the 1000-km distance that spans between the Cabot Strait and the Grande-Rivière-Blanche (Québec) represents the longest estuarine distance among all sampled localities. Historically, CPUE data in the Gulf of St. Lawrence have several orders of magnitude below those reported on the Atlantic shore of Nova-Scotia, probably as a result of less-favorable conditions encountered by eels in the Gulf (Dutil et al. 2009). Thus, the very high RNA/DNA ratio we observed for the Gaspésie glass eels could reflect physiological stress encountered in the St. Lawrence estuary, perhaps associated with long osmoregulation acclimation during the stabulation period in the estuary (McGovern and McCarthy 1992).

Overall then, it seems plausible that oceanic conditions might be more favorable to glass eel colonizing the middle of their range, resulting in overall higher survival and recruitment in this part of the species’ range. In contrast, glass eel that disperse at either the northern or southern extremities of the distribution range may encounter physiologically more stressful conditions leading to reduced survival and recruitment. This hypothesis is supported by the predominance of glass eels characterized by relatively low RNA–DNA ratios and high condition factor in the middle part, in contrast to the predominance of glass eels with high RNA–DNA ratios and low condition factors at both the southern and northern extremities of the distribution range. Differential selective pressures associated with variable environmental conditions being encountered could then result in genetic differences between glass eels from different locations through the process of spatially varying selection (Levene 1953) as documented by Gagnaire et al. (2012). Moreover, although the analysis of neutral genetic markers has still not refuted the null hypothesis of panmixia for American eel (Avise et al. 1986; Wirth and Bernatchez 2003), common rearing experiment of glass eels from two different locations that were analyzed in this study (Grande-Rivière-Blanche, Gaspésie, Québec and Mira River, Cap Breton, Nova Scotia) revealed quantitative genetic differences in growth between glass eels from these two localities (Côté et al. 2009). On the other hand, Wang and Tzeng (2000) hypothesized that individual growth could influence patterns of dispersal and Edeline et al. (2004) suggested that different energetic status could influence migratory behavior and habitat selection. Thus, an additional (not exclusive) explanation to differential selection might involve differential dispersal stemming directly from the Sargasso Sea associated with physiological status, which could also result in local genetic differences within an otherwise panmictic species (Williams et al. 1973; Koehn and Williams 1978).

Latitudinal variation in energetic status could also be related to catadromy rates (the percentage of individuals colonizing freshwater habitats) that vary throughout the species’ range. As a general tendency, it appears that a larger contingent of eels will settle in saltwater habitats at higher latitudes, possibly due to a generally higher productivity in coastal marine environments versus freshwater habitats at northern latitudes (Daverat et al. 2006; Vélez-Espino and Koops 2009). Namely, Edeline (2007) proposed that the “freshwater vs. saltwater” dilemma for the eel could be thought of as an evolutionary stable conditional strategy model, whereby differential migration to either freshwater or marine (brackish) habitats will depend on individual energetic status. However, the hypothesis that catadromous behavior depends on energetic status in American eel must still be rigorously tested.

### The influence of DNA content on the observed RNA/DNA ratios

The differences observed between DNA concentration measured in glass eels were mainly due to middle range localities featuring the highest DNA contents (Table 2). Bishop et al. (2000) have previously described the first growth phase (Ia) of leptocephalus larvae (during which they elongate to nearly their full glass eel size) as a period of intense cell proliferation. Since DNA content is proportional to the number of cells, this phase could be important in influencing the total DNA content of the future glass eels. It is also noteworthy that the results of Wang and Tzeng (1998) suggested that glass eels collected from the middle distribution range were also characterized by a faster growth rate during the leptocephalus stage. Although hypothetical, it is plausible that differential growth rate during the leptocephalus stage could contribute to the observed latitudinal differences in DNA content and therefore on patterns of RNA/DNA ratios. Finally, it is also possible that variable times at arrival per se could have influenced the DNA content of glass eels. For instance, in the Japanese eel, Kawakami et al. (1999) noticed a slight but significant temporal increase of DNA content among successive waves of glass eels arriving at a single sampling site. In the present study, the date of capture could not be standardized but always corresponded to the first wave of glass eels arrival at each location. Clearly then, many factors must be considered in interpreting RNA/DNA ratios as a proxy for energetic status in species such as the American eel whose recruitment occurs at different times of the year depending on latitudes, and may last between two to six months, depending on year (Overton and Rulifson 2009; Sullivan et al. 2009). Yet, there is little doubt that the patterns of RNA–DNA ratios and condition factor we quantified in this study reflect pronounced differences in physiological status among glass eels entering freshwater at different latitudes and the study of Gagnaire et al. (2012) suggests that this is accompanied by differences at genes under the effect selection.
